# Supplementary material for: A comparison of national seasonal influenza treatment guidelines across the Asia Pacific region
Source: PLOS Glob Public Health. 2025 Apr 28;5(4):e0004468. doi: 10.1371/journal.pgph.0004468 (PMC12036931; doi:10.1371/journal.pgph.0004468)
Supplement: S1 Table — (DOCX) [file pgph.0004468.s001.docx]

## S1 Table. Data Extraction criteria and questions

| Domain |  | | Answer format |
| --- | --- | --- | --- |
| Availability,  Scope and  Purpose | Is an official national guideline in place? | | Yes/No/Unknown |
|  | Is it publicly available? | | Yes/No |
|  | Year of Publication (Gregorian calendar) | | Year (YYYY) |
|  | Guideline Title | | Short text answer/Unspecified |
|  | Influenza type | | Seasonal/Pandemic/Zoonotic/Unspecified |
|  | Is the healthcare setting or context specified? | | Yes/No – Short text answer |
|  | Have the clinical outcomes for which the guidelines are based been described (e.g. reduce risk hospitalisation, reduce risk mortality, reduced symptom duration)? | | Yes/No |
|  | What is the indication for treatment? | | Short text answer |
|  | Are high risk groups defined? | | Yes/No |
|  | What are the high-risk groups? | | Long text answer |
|  | How is complicated/severe influenza defined? | | Short text answer |
|  | How is uncomplicated/mild influenza defined? | | Short text answer |
| Stakeholder  Involvement | What is the name of the body/institution who produced the guideline? | | Short text answer/Unspecified |
|  | Are the details of guideline authors provided? | | Yes/No |
|  | Are the guideline target users stated? E.g. clinicians | | Yes/No |
| Rigour of  development | Are guideline formulation  methods included? | | Yes/No |
|  | Is a detailed evidence review included? | | Yes/No |
|  | Is the evidence graded/strength recommendation provided? | | Yes/No |
| Clarity of Presentation | Are the following antivirals recommended? | Oseltamivir | Yes/Unspecified/No (recommended against use) |
|  |  | Zanamivir |  |
|  |  | Laninamivir |  |
|  |  | Peramivir |  |
|  |  | Amantadine |  |
|  |  | Rimantadine |  |
|  |  | Baloxavir |  |
|  |  | Favipiravir |  |
|  |  | Umifenovir |  |
|  |  | Other |  |
|  | Is a diagnostic requirement stated? | | Short text answer |
|  | Is a symptom window for  treatment given? | | Short text answer |
|  | Is the dose specified? | | Yes/Unspecified |
|  | Is the route of administration specified? | | Yes/Unspecified |
|  | Is safety/toxicity information included? | | Yes/Unspecified |
|  | Is an order of recommendation provided? | | Yes/Unspecified/Not applicable |
|  | Cost analysis | | Yes/Unspecified |
| Applicability | Regulatory status of antiviral: is the recommended antiviral approved by the national drug regulatory authority? | | Yes/No/Unspecified |
|  | Is the guideline publicly  available for target users | | Yes/No/Unspecified |
| Editorial  independence | Competing interests declared | | Yes/No |
