# Supplementary material for: A comparison of national seasonal influenza treatment guidelines across the Asia Pacific region
Source: PLOS Glob Public Health. 2025 Apr 28;5(4):e0004468. doi: 10.1371/journal.pgph.0004468 (PMC12036931; doi:10.1371/journal.pgph.0004468)
Supplement: S2 Table — (DOCX) [file pgph.0004468.s002.docx]

## S2 Table: Summary of Existing National Guidelines from World Health Organisation South-East Asia Region Countries and Areas

| Country (and WHO) | WHO | India | Myanmar | Nepal | Sri Lanka | Thailand |
| --- | --- | --- | --- | --- | --- | --- |
| Publicly available | Yes | Yes | Yes | Yes | Yes | No |
| Publication Year | 2021 | 2019 | 2017 | 2016 | 2015 | 2023 |
| Institution | Guideline Development Group (WHO) | Ministry of Health and Family Welfare Government of India | Ministry of Health and Sports Myanmar, National Institute for Communicable Diseases, Central Level Advisory Committee, Department of Disease Control | Ministry of Health  Epidemiology and Disease Control Division (EDCD) | Ministry of Healthcare & Nutrition, Ministry of Health and Indigenous Medicine | Ministry of Public Health |
| Authors listed | Yes | No | No | Yes | No | No |
| Guideline Title | Guidelines for the clinical management of severe illness from influenza virus infections | Clinical Management Protocol for Seasonal Influenza | Management Guideline for Seasonal Influenza A(H1N1)pdm 2009 infection | Infectious Disease Control Guidelines | Revised Summary Guidelines for Clinical Management and Laboratory Investigation of Patients with Seasonal Influenza Virus Infection | Recommendations for Treating Influenza Patients for Medical and Public Health Personnel |
| Influenza type | S, P, Z | S | S | S, P, Z | S | S, P, Z |
| Target users stated | Clinicians, health care providers, policymakers, health managers and health facility administrators | No | Medical officers/physicians, Emergency medical officers | No | No | Medical and public health personnel |
| Guideline formulation methods stated | Yes | No | Yes | No | No | No |
| Evidence reviewed | Yes | No | No | No | No | No |
| Evidence graded | Yes | No | No | No | No | No |
| Healthcare setting/ context | Yes - all levels of healthcare system | Yes - all level health facilities, government and private | Yes - Hospital (implied by discussing wards) | No | Yes - Hospital outpatient settings | No |
| Clinical outcomes stated | Yes | No | Yes | Yes | No | No |
| Treatment indication | Suspected or confirmed influenza virus infection and/ or at risk of severe illness | Category B and C | Category Bii and C | Confirmed or suspected influenza who: - is hospitalized; - has severe, complicated, or progressive illness; or - is at higher risk for influenza complications. | Hospitalised patients who are - Severe/complicated cases or have signs of progression of the disease  -conditional for high-risk groups – on a case-by-case basis | 1. Severe 2. Uncomplicated at high risk 3. Mild cases (conditional - can be considered if within 48 hours) |
| High risk group | P, C, I, O, A, B, H, Co, Hi/Imm | P, C, O, Co, HI/Imm, B | P, Co, Hi/Imm | O, Co, C* (Malnourished), P, B, HI/Imm | I, O, P, Co, HI/Imm | B (BMI > 30), P, I, O* (>60 years), Co, HI/Imm, A* (<18 years) |
| Severe/ Complicated influenza definition | “Influenza virus infection illness that would lead to hospitalization. This includes patients with clinical syndromes such as a) severe pneumonia, ARDS; b) sepsis, multiorgan failure or shock; c) exacerbation or complications associated with chronic diseases, such as diabetic crises, asthma attack, COPD exacerbation, acute heart failure or acute renal failure”. | Category C: “Uncomplicated PLUS one of: 1. Breathlessness 2. Haemoptysis 3. Altered mental status 4. Somnolence and Poor feeding (in children) 5. Seizures 6. Decreased urine output 7. Persistence or worsening of initial symptoms beyond 72 hours 8. Worsening of underlying chronic conditions or 1. Tachypnoea 2. SpO2<90% 3. Hypotension 4. Reduced urine output 5. Cyanosis” | “Category C: Category A PLUS breathlessness, chest pain, drowsiness, hypotension, cyanosis, tachypnoea, decreased oxygen saturations, patchy opacities on CXR” | “Mild cases with complications: a. Bronchitis, bronchiolitis b. Primary influenza viral pneumonia c. Secondary viral pneumonia- d. GI symptoms- watery diarrhoea more frequently described during influenza A H1N1 and H5N1 than seasonal infection e. Neurological- encephalitis, transverse myelitis, acute necrotizing encephalitis, GBS f. Cardiovascular- ECG abnormalities, myocarditis, pericarditis g. Otitis media, conjunctivitis h. Myositis, myoglobinuria and ARF” | “Severe/complicated or progressive illness (e.g., shortness of breath/dyspnoea, tachypnoea, hypoxia), signs of pneumonia, CNS involvement, severe dehydration, Signs of organ failure, exacerbation of underlying chronic disease” | “ILI cases with - suspected pneumonia from symptoms or CXR - SpO2 at room air less than 95% in patients who must use oxygen - abnormal depression or neurological symptoms - Eating so little that there is dehydration - have complications or other serious symptoms” |
| Mild /uncomplicated influenza definition | “Cases with sudden onset of cough, headache, muscle and joint pain, severe malaise, sore throat and a runny nose, with or without fever” | “Category A= Mild fever plus cough/sore throat with or without body ache, headache, vomiting, diarrhoea. Category B (Category A, uncomplicated, PLUS severe symptoms): such as high-grade fever and severe sore throat, or high-risk group.” | “Category A: Mild fever plus cough/sore throat with or without body ache, headache, vomiting, diarrhoea.  Category Bi = Category A PLUS high fever and severe sore throat, or (Bii) = mild ILI with high-risk co-morbidities” | “Flu symptoms- acute onset of fever, cough, headache, coryzal symptoms, sore throat, myalgia” | “Cases presenting with acute febrile illness, with URTI from ILI to pneumonia (cough, sore throat, rhinorrhoea, headache, muscle pain and malaise but no shortness of breath and no dyspnoea. Patients may present with some or all of these symptoms: gastrointestinal symptoms such as diarrhoea and/or vomiting may be present especially in children, but without evidence of dehydration” | “ILI: Fever >38 C PLUS cough and/or sore throat (may be other symptoms such as stuffy nose, runny nose, aches, vomiting, diarrhoea)” |
| Diagnostic requirements | Conditional: if available and result <24 hours | Conditional: category C | Conditional: Category B(ii) & Category C | Treatment decisions should not wait for laboratory confirmation of H1N1 infection | Diagnostic samples to be collected based on clinical judgement in hospitalised patients only, conditionally based on testing availability | Unspecified |
| Oseltamivir | Yes | Yes | Yes | Yes | Yes | Yes |
| Zanamivir | No | Unspecified | Unspecified | Yes | Unspecified | Unspecified |
| Peramivir | No | Unspecified | Unspecified | Unspecified | Unspecified | Unspecified |
| Laninamivir | No | Unspecified | Unspecified | Unspecified | Unspecified | Unspecified |
| Amantadine | No | Unspecified | Unspecified | Unspecified | Unspecified | Unspecified |
| Baloxavir | Unspecified | Unspecified | Unspecified | Unspecified | Unspecified | Unspecified |
| Favipiravir | Unspecified | Unspecified | Unspecified | Unspecified | Unspecified | Yes |
| Umifenovir | Unspecified | Unspecified | Unspecified | Unspecified | Unspecified | Unspecified |
| Other | Unspecified | Unspecified | Unspecified | Unspecified | Unspecified | Unspecified |
| Corticosteroids | No | No | No | Unspecified | Unspecified | Unspecified |
| Order of recommendation | Not applicable | Not applicable | Not applicable | No | Not applicable | Yes |
| Symptom window | "as soon as possible" | "As early as possible, aim for within 48 hours" | "As soon as possible" | "As soon as possible, aim for within 48 hours" | "For immediate initiation" | "As soon as possible" "Treatment in uncomplicated patients may be considered if administered within 48 hours” |
| Dose Specified | No | Yes | Yes | Yes | Yes | Yes |
| Safety/toxicity information | Yes | Yes | No | Yes | Yes | Yes |
| Regulatory status | Not applicable | No | No | No | No | No |
| Cost Effectiveness Analysis | No | No | No | No | No | No |
| Competing Interest Declaration | Yes | No | No | No | No | No |

***Abbreviations:*** *ARDS: Acute Respiratory Distress Syndrome; ARF: Acute Renal Failure; CNS: Central Nervous System; COPD: Chronic Obstructive Pulmonary Disease; CXR: Chest X-Ray; ECG: Electrocardiogram; GBS: Guillain-Barré Syndrome; ILI: Influenza-like Illness; SARI: Severe Acute Respiratory Illness; URTI: Upper Respiratory Tract Infection; WHO: World Health Organisation.*

*High Risk Groups: A: people younger than 19 years of age on long-term aspirin- or salicylate-containing medications; B: people with a body mass index (BMI) of 40 or higher; C: Children <5 years; Co: Co-morbidities (cardiac, renal, lung, liver, neurological/neurodevelopmental, haematological, endocrine, metabolic); H: Healthcare workers; H/Imm: HIV/Immunocompromised; Ho: No fixed abode; N: Nursing home or chronic care facility residents; I: Infants <2 years old; O: Older people ≥ 65; P: Pregnant women and women up to 2 weeks postpartum.*

*Influenza-Type: S: Seasonal; P: Pandemic, Z: Zoonotic*

**Conditional recommendation*
