# Supplementary material for: A comparison of national seasonal influenza treatment guidelines across the Asia Pacific region
Source: PLOS Glob Public Health. 2025 Apr 28;5(4):e0004468. doi: 10.1371/journal.pgph.0004468 (PMC12036931; doi:10.1371/journal.pgph.0004468)
Supplement: S3 Table — (DOCX) [file pgph.0004468.s003.docx]

## S3 Table. Summary of Existing National Guidelines from World Health Organisation Western Pacific Region Countries and Areas

| Country | Australia | China | Hong Kong | Japan | Kiribati | Malaysia | South Korea | Taiwan | Vietnam |
| --- | --- | --- | --- | --- | --- | --- | --- | --- | --- |
| Publicly available | No | No | Yes | Yes | Yes | Yes | Yes | Yes | No |
| Publication Year | 2019 | 2019 | 2022 | 2019 | 2011 | 2023 | 2012 | 2017 | 2011 |
| Body/Institution | Therapeutic Guidelines Australia | General Office of the National Health Commission General Office of the State Administration of Traditional Chinese Medicine | Task Force on Clinical Management on Infection (TFCM), Central Committee on Infectious Diseases and Emergency Response (CCIDER) | Japanese Society of Infectious Diseases | Secretariat of the Pacific Community (SPC), Ministry of Health and Medical Services (MHMS), Kiribati Secretariat of the Pacific Community Noumea, New Caledonia | Primary Health Branch Family Health Development Division, Ministry of Health Malaysia | The Korean Society of Infectious Diseases / Korean Society for Chemotherapy | Ministry of Health, Department of Disease Control | Ministry of Health |
| Authors listed | No | No | No | Yes | Yes | No | Yes | No | No |
| Guideline Title | Influenza Therapeutic Guidelines | Guidelines for diagnosis and treatment of influenza (2019 version) | Use of neuraminidase inhibitors in out-patient settings | Recommendations of the Japanese Society of Infectious Diseases | Clinical case management guideline for influenza, including pandemic influenza A(H1N1) 2009 | Guidelines for Handling Influenza in Health Clinic | Clinical Practice Guideline for Antiviral Treatment and Chemoprophylaxis of Seasonal Influenza | Practical Guideline for Prevention and Control of Seasonal Influenza | Decision on the Promulgating of Guidelines for the Diagnosis and Treatment of Seasonal Flu |
| Influenza type | S, Z | S, P, Z | S, P, Z | S | S, P, Z | S, P, Z | S | S | S |
| Target users | No | No | Doctors | Physicians | ‘Health-care workers, medical assistants’ | ‘Doctors, health personnel who manage triage in clinic settings’ | ‘Doctors in all medical specialties who provide care for influenza patients’ | Implies all personnel involved in seasonal flu outbreak control, surveillance, testing, case management. | Ministry of health ministers listed, Directors of Hospitals and Institutes with beds under the Ministry of Health, Provincial and City Directors of Health Departments |
| Guideline formulation methods stated | No | No | No | Yes | Yes | No | Yes | Yes | No |
| Evidence reviewed | No | No | No | Yes | No | No | Yes | Yes | No |
| Graded recommendations | No | No | No | No | No | No | Yes | No | No |
| Healthcare setting/ context | No | No | Yes -Outpatient settings | Yes – all medical settings | Yes –  Outpatient medical duty stations | Yes –  Outpatient health clinics | Yes – All medical settings | Yes –  All medical settings | Yes – All level medical settings, government and private |
| Clinical outcomes stated | Yes | Yes | Yes | Yes | No | No | Yes | Yes | No |
| Treatment indication | Offer to patients with established complications OR patients requiring hospitalisation OR patients with moderate-severity or high-severity community-acquired pneumonia during the influenza season. Consider in high-risk individuals, residents of an aged-care facility, individuals with high risk HH contacts | Individuals with severe illness or high-risk factors for severe influenza | Any patient with confirmed or suspected influenza who: (i) has severe, complicated, or progressive illness; or (ii) is at higher risk for influenza complications | High risk groups, hospitalised, severe, and progressive symptoms, uncomplicated and complicated cases within 48 hours of symptom onset, healthcare workers, patients with HH contacts who are high risk for complications from influenza | ILI + high risk, SARI, or ILI + progression/ deterioration of disease (as defined in guideline) | ILI PLUS high risk or moderate/severe influenza | Meets ILI definition, hospitalised, severe or with complications, worsening clinical course, high risk groups, and also low risk individuals <48 hours, patient in contact with high-risk groups | Unspecified | Complicated flu, consider early treatment in high-risk groups |
| High risk group | C, O, P, HI/Imm, B* (BMI >30) Co, N, H, Aboriginal and Torres Strait Islander people of any age | C, O, Co, Hi/Imm, B* (>30), P | I, O, Co, Hi/Imm, P, A, B, N | C, I, O, Co, Hi/Imm, P, A, B, N | Co, Hi/Imm, A, O, P, C, I | Co, P, O, Hi/Imm, O, C, I | O,I, Co, Hi/Imm, P, C, B, N | O, I, P, Hi/Imm, Co, B (>=30) | Co, Hi/Imm, A, C, P, obese children |
| Severe influenza definition | Cases that require ventilatory or haemodynamic support | Continuous high fever for > 3 days, accompanied by severe cough, coughing up purulent sputum, bloody sputum, or chest pain; 2. Rapid respiratory rate, dyspnoea, cyanosis of lips; 3. Mental changes: slow reaction, drowsiness, restlessness, convulsions, etc.; 4. Severe vomiting, diarrhoea, and signs of dehydration; 5. Combined with pneumonia; 6. Significant aggravation of the original underlying disease; 7. Other clinical conditions requiring hospitalization. (2) Those who have any of the following conditions are considered critical cases: 1. Respiratory failure; 2. Acute necrotizing encephalopathy; 3. Septic shock; 4. Multiple organ dysfunction; 5. Other serious clinical conditions requiring intensive care. | Unspecified | Unspecified | SARI defined as ILI PLUS at least one of: • fast breathing • infiltrate on chest X-ray. But because X-rays are not available in the Outer Islands, HCWs need to look out for one or more of the following respiratory signs and symptoms: – difficulty in breathing and chest heaving; – use of accessory muscles, supra-clavicular recession, tracheal tug (mainly in adults and older children); – lower-chest in-drawing, sternal recession or noisy breathing when calm, flaring of alae nasi (especially in children); – inability to complete a sentence without stopping for air; – feeling of suffocation; – new chest-signs upon auscultation, including crepitations/rhonchi, decreased breath sound or silent chest, dullness to percussion. | ILI PLUS Respiratory impairment/evidence clinical dehydration or shock/altered GCS, other clinical concerns. Signs and symptoms of moderate to severe influenza 1. Respiratory difficulty: Shortness of breath, rapid breathing or purple or blue discoloration of lips 2. Coughing out blood or blood streaked sputum 3. Persistent chest pains 4. Persistent diarrhoea and /or vomiting 5. Fever persisting beyond 3 days or recurring after 3 days 6. Abnormal behaviour, confusion , less responsive , convulsion 7. Dizziness when standing and/ or reduced urine production | “influenza corresponding to the definition of influenza-like illness (ILI; sudden onset of fever and cough or sore throat) and presenting at least one of the following clinical presentations: - Dyspnea, tachypnea, or hypoxia - Radiological signs of LRTI disease - CNS involvement (e.g., encephalopathy, encephalitis) - Severe dehydration – Acute renal failure – Septic shock - Exacerbation of underlying chronic disease, including asthma, COPD, chronic hepatic or renal insufficiency, diabetes mellitus, or other cardiovascular conditions - Any other influenza-related condition or clinical presentation requiring hospital admission | “Complications: viral pneumonia, secondary bacterial pneumonia, CNS manifestation, cardiac manifestation, exacerbation of co-morbidities. Signs of deterioration: difficulty breathing, tachypnoea, cyanosis, altered GCS, hypotension, sustained high fever.” | “Clinical syndrome of flu PLUS Lung damage with clinical manifestations of respiratory failure (rapid breathing, difficulty breathing, decreased SpO2, decreased PaO2) and/or: secondary complications such as sinusitis, pneumonia due to bacterial superinfection, septic shock, multiple organ failure. Or + There are signs of worsening of accompanying chronic diseases (lung disease, liver disease, kidney failure, diabetes, cardiovascular disease, blood disease)” |
| Mild influenza definition | Unspecified | Flu like symptoms without complications | Unspecified | Unspecified | ILI definition: ‘sudden onset of fever (measured) ≥38°C AND at least one of the following symptoms: cough and/or sore throat. ILI case definition is met BUT there is no shortness of breath or dyspnoea. • Diarrhoea and/or vomiting may be present with no sign of dehydration. | ILI: History of high fever with temperature > 38°C and cough with onset within the last ten (10) days | ILI definition: sudden onset of fever with coughing or sore throat. | ILI: Sudden onset, fever (ear temperature ≧38°C or above) and respiratory symptoms. | Fever (usually above 38 degrees C), generalized muscle aches and one of the following respiratory symptoms: sore throat, sneezing, stuffy nose, runny nose, cough, difficulty breathing. |
| Diagnostic requirement | Discusses possible tests, does not provide recommendation | Conditional: Yes, unless causes delay in treatment | Unspecified | Unspecified | No – due to limited local availability | Not required | Doesn’t specify discusses techniques, and that confirmatory testing is not necessary during epidemics | Conditionally recommends - do not wait for results to start treatment | Lists options, does not set conditions or recommendations |
| Oseltamivir | Yes | Yes | Yes | Yes | Yes | Yes | Yes | Yes | Yes |
| Zanamivir | Yes | Yes | Unspecified | Yes | Unspecified | Unspecified | Yes | Yes | Yes |
| Peramivir | Yes | Yes | Unspecified | Yes | Unspecified | Unspecified | Yes | Yes | Unspecified |
| Laninamivir | Unspecified | Unspecified | Unspecified | Yes | Unspecified | Unspecified | Unspecified | Unspecified | Unspecified |
| Amantadine | Unspecified | No | Unspecified | Unspecified | Unspecified | Unspecified | Yes* | No | Unspecified |
| Rimantadine | Unspecified | No | Unspecified | Unspecified | Unspecified | Unspecified | Unspecified | No | Unspecified |
| Baloxavir | Unspecified | Unspecified | Unspecified | Yes* | Unspecified | Unspecified | Unspecified | Unspecified | Unspecified |
| Favipiravir | Unspecified | Unspecified | Unspecified | No* | Unspecified | Unspecified | Unspecified | Unspecified | Unspecified |
| Umifenovir | Unspecified | Yes | Unspecified | Unspecified | Unspecified | Unspecified | Unspecified | Unspecified | Unspecified |
| Other | Unspecified | Herbal remedies | Unspecified | Unspecified | Unspecified | Unspecified | Unspecified | Unspecified | Unspecified |
| Corticosteroids | No | Unspecified | Unspecified | Unspecified | Unspecified | Unspecified | Unspecified | Unspecified | Unspecified |
| Order of recommendation | No | No | Not applicable | No | Not applicable | Not applicable | No | No | No |
| Symptom window | “Offer treatment to indicated groups regardless of the duration of symptoms” | “As early as possible, target <48 hours” “Uncomplicated low risk patients may be considered within 48 hours, if risks and benefits considered” | “as soon as possible” aim <48 hours | Target <48 hours | Target <48 hours | Target <48 hours | Target <48 hours | Target <48 hours | “As soon as possible” |
| Dose Specified | Yes | Yes | Yes | Yes | Yes | Yes | Yes | No | Yes |
| Safety/Toxicity information | No | No | Yes | Yes | Yes | No | Yes | No | No |
| Regulatory Status Stated | No | Yes | No | Yes | No | No | Yes | Yes | No |
| Cost effectiveness analysis | No | No | No | No | No | No | No | No | No |
| Competing interest declaration | No | No | No | No | No | No | Yes | No | No |

***Abbreviations:*** *CNS: Central Nervous System; LRTI: Lower Respiratory Tract Infection; HH: Household; ILI: Influenza-like Illness; SARI: Severe Acute Respiratory Illness; WHO: World Health Organisation.*

*High Risk Groups: A: people younger than 19 years of age on long-term aspirin- or salicylate-containing medications; B: people with a body mass index (BMI) of 40 or higher; C: Children <5 years; Co: Co-morbidities (cardiac, renal, lung, liver, neurological/neurodevelopmental, haematological, endocrine, metabolic); H: Healthcare workers; H/Imm: HIV/Immunocompromised; Ho: No fixed abode; N: Nursing home or chronic care facility residents; I: Infants <2 years old; O: Older people ≥ 65; P: Pregnant women and women up to 2 weeks postpartum.*

*Influenza-Type: S: Seasonal; P: Pandemic, Z: Zoonotic*

**Conditional recommendation*
