# Supplementary material for: A comparison of national seasonal influenza treatment guidelines across the Asia Pacific region
Source: PLOS Glob Public Health. 2025 Apr 28;5(4):e0004468. doi: 10.1371/journal.pgph.0004468 (PMC12036931; doi:10.1371/journal.pgph.0004468)
Supplement: Attachment — Submitted filename: Response to Reviewers.docx [file pgph.0004468.s005.docx]

**Response to Reviewers**

Reviewer's Responses to Questions

**Comments to the Author**

1. Does this manuscript meet PLOS Global Public Health’s [publication criteria](https://track.editorialmanager.com/CL0/https:%2F%2Fjournals.plos.org%2Fglobalpublichealth%2Fs%2Fcriteria-for-publication/1/010f019385b3da75-772414e2-89f4-4fb5-9b8a-63e99e1de830-000000/YFbdACTZ9GNeJdrcsmqrCs95reFVStsBLs4JX33UndE=187)? Is the manuscript technically sound, and do the data support the conclusions? The manuscript must describe methodologically and ethically rigorous research with conclusions that are appropriately drawn based on the data presented.

Reviewer #1: Yes

2. Has the statistical analysis been performed appropriately and rigorously?

Reviewer #1: N/A

3. Have the authors made all data underlying the findings in their manuscript fully available (please refer to the Data Availability Statement at the start of the manuscript PDF file)?

The [PLOS Data policy](https://track.editorialmanager.com/CL0/https:%2F%2Fjournals.plos.org%2Fglobalpublichealth%2Fs%2Fdata-availability/1/010f019385b3da75-772414e2-89f4-4fb5-9b8a-63e99e1de830-000000/OopItQCDHT_OsGaIDGFh-0xYkWxXoCy5afOTj6LLzZc=187) requires authors to make all data underlying the findings described in their manuscript fully available without restriction, with rare exception. The data should be provided as part of the manuscript or its supporting information, or deposited to a public repository. For example, in addition to summary statistics, the data points behind means, medians and variance measures should be available. If there are restrictions on publicly sharing data—e.g. participant privacy or use of data from a third party—those must be specified.

Reviewer #1: Yes

4. Is the manuscript presented in an intelligible fashion and written in standard English?

PLOS Global Public Health does not copyedit accepted manuscripts, so the language in submitted articles must be clear, correct, and unambiguous. Any typographical or grammatical errors should be corrected at revision, so please note any specific errors here.

Reviewer #1: Yes

5. Review Comments to the Author

Please use the space provided to explain your answers to the questions above. You may also include additional comments for the author, including concerns about dual publication, research ethics, or publication ethics. (Please upload your review as an attachment if it exceeds 20,000 characters)

Reviewer #1: Congratulations for this relevant and well written manuscript. Few comments:
-Line 54: statement only correct for NH recommendation, not for SH. Pls add sentence for SH

Thank you – we have revised this to detail NH/SH differences more clearly.

Line 59: mention here or somewhere else in the manuscript just as context that the mRNA or sa-mRNA based vaccine candidates could become a game changer in Flu management. They will have the advantage that one can wait longer to decide on strains as the manufacturing process is quicker, hence having potentially better matches. Also, it seems that most of those include also Neuraminidase Ag for improved protection

Thank you – we appreciate this suggestion. We have added this in the introduction.

107ff: check sentences

Thank you – we have adjusted the wording, so it is more cohesive.

244: which 6 countries? Please list

Thank you – now listed.

Figure 2: contacted countries = 38. Responders 33. and the other 5?

Thank you – 38 countries were contacted, 24 countries did not respond with a definite answer, 9 did respond to say they do not have a national guideline in place, 5 countries responded with statements about the influenza guidelines in place and attached or linked documents (n=11) to influenza guidelines, that were assessed by the authors for eligibility.

Discussion: good, but "so what"? What do you recommend as outcome of your research? for example, one recommendation could be that WHO includes in their recommendations details on Favipiravir , Baloxavir. Another recommendation could be that WHO regional offcies calls for a Flu summit where countries can share best practices

Thank you – we appreciate this point. We have made clearer recommendations. In addition, we have acknowledged that new WHO influenza clinical management guidelines have been published after this review has been conducted, therefore reinforcing the importance of a timely regional and domestic reviewing of the evidence, sharing best practices across the region as you have noted, reviewing WHO recommendations and adapting and updating national guidelines.

6. PLOS authors have the option to publish the peer review history of their article ([what does this mean?](https://track.editorialmanager.com/CL0/https:%2F%2Fjournals.plos.org%2Fglobalpublichealth%2Fs%2Feditorial-and-peer-review-process%23loc-peer-review-history/1/010f019385b3da75-772414e2-89f4-4fb5-9b8a-63e99e1de830-000000/IcAKjLih3s5XJTFXAJK8lUMr1ucHgsRGMTNCbJGBCTw=187)). If published, this will include your full peer review and any attached files.


**Do you want your identity to be public for this peer review?** If you choose “no”, your identity will remain anonymous but your review may still be made public.


For information about this choice, including consent withdrawal, please see our [Privacy Policy](https://track.editorialmanager.com/CL0/https:%2F%2Fwww.plos.org%2Fprivacy-policy/1/010f019385b3da75-772414e2-89f4-4fb5-9b8a-63e99e1de830-000000/CO_2wl1CXsGpMewpz1CxZyJbCXVUHmturP1tEYnpbnc=187).

Reviewer #1: No
