# Supplementary material for: A comparison of national seasonal influenza treatment guidelines across the Asia Pacific region
Source: PLOS Glob Public Health. 2025 Apr 28;5(4):e0004468. doi: 10.1371/journal.pgph.0004468 (PMC12036931; doi:10.1371/journal.pgph.0004468)
Supplement: Attachment — Submitted filename: Response_to_Reviewers_auresp_2.docx [file pgph.0004468.s006.docx]

**Response to Reviewers**

Reviewer's Responses to Questions

**Comments to the Author**

1. If the authors have adequately addressed your comments raised in a previous round of review and you feel that this manuscript is now acceptable for publication, you may indicate that here to bypass the “Comments to the Author” section, enter your conflict of interest statement in the “Confidential to Editor” section, and submit your "Accept" recommendation.

Reviewer #1: All comments have been addressed

Reviewer #2: All comments have been addressed

2. Does this manuscript meet PLOS Global Public Health’s [publication criteria](https://track.editorialmanager.com/CL0/https:%2F%2Fjournals.plos.org%2Fglobalpublichealth%2Fs%2Fcriteria-for-publication/1/010f019511eb2add-56bc6c6f-9c2b-4bcf-9252-bc00d3e81588-000000/RwayBf6Y8j3qq1q6-ZyKcnjfZh4TDUJI3xetuAln_R8=198)? Is the manuscript technically sound, and do the data support the conclusions? The manuscript must describe methodologically and ethically rigorous research with conclusions that are appropriately drawn based on the data presented.

Reviewer #1: Yes

Reviewer #2: Yes

3. Has the statistical analysis been performed appropriately and rigorously?

Reviewer #1: N/A

Reviewer #2: Yes

4. Have the authors made all data underlying the findings in their manuscript fully available (please refer to the Data Availability Statement at the start of the manuscript PDF file)?

The [PLOS Data policy](https://track.editorialmanager.com/CL0/https:%2F%2Fjournals.plos.org%2Fglobalpublichealth%2Fs%2Fdata-availability/1/010f019511eb2add-56bc6c6f-9c2b-4bcf-9252-bc00d3e81588-000000/DLR71BJleLiusZU4WUsXTXLxfi2Rf1bIEMFCc9lkXE4=198) requires authors to make all data underlying the findings described in their manuscript fully available without restriction, with rare exception. The data should be provided as part of the manuscript or its supporting information, or deposited to a public repository. For example, in addition to summary statistics, the data points behind means, medians and variance measures should be available. If there are restrictions on publicly sharing data—e.g. participant privacy or use of data from a third party—those must be specified.

Reviewer #1: Yes

Reviewer #2: Yes

5. Is the manuscript presented in an intelligible fashion and written in standard English?

PLOS Global Public Health does not copyedit accepted manuscripts, so the language in submitted articles must be clear, correct, and unambiguous. Any typographical or grammatical errors should be corrected at revision, so please note any specific errors here.

Reviewer #1: Yes

Reviewer #2: Yes

6. Review Comments to the Author

Please use the space provided to explain your answers to the questions above. You may also include additional comments for the author, including concerns about dual publication, research ethics, or publication ethics. (Please upload your review as an attachment if it exceeds 20,000 characters)

Reviewer #1: Thanks for including the recommendations and also for highlighting the new WHO recommendation.

Reviewer #2: This is an excellent study that uses appropriate methods to provide a comprehensive review of guidelines. The study team also nicely addressed the previous peer-reviewer comments. I have minor suggestions which I hope will help the presentation.

1) Please clarify how it was determined that there was “no guideline in place” and what “unknown” means in Table 1. It would be helpful to first indicate approach in the methods section (and how these two elements would thus be defined, and then refer to the results as currently included in lines 238-248).

2) It was unclear what this means, is it because no guidelines were in place? “We do not include Bangladesh or Palau in analysis of national guidelines for the purpose of this report.”

3) This statement in the discussion was interesting an important. It would be helpful to provide some context and further explanation in the discussion– (a) are these elements generally discussed in influenza guidelines and should they be discussed and/or included in treatment guidelines: “Discussion about cost, availability, and accessibility was rarely included in guidelines.”; and (b) could authors elaborate on the point made in line 480 (“Some policy papers show significant variability in distribution and availability of oseltamivir and other influenza treatment resource gaps domestically especially in rural areas in Southeast Asia.”), and comment on potential considerations if/how access/availability might shape variability in guidelines or whether guidelines exist within countries?

Thank you greatly for agreeing to and taking the time to review our paper.

1. Thank you for flagging this – it is something we have now made clearer in the methods section. Where a national prominent policymaker has replied to formally say that a national level guideline does not exist, we have used ‘no guideline in place’. Thus we are able to confirm the absence of a clinical guideline (according to our inclusion criteria, ie any basic document that would guide clinical practitioners decision-making sufficiently). For countries/territories for whom we did not receive a response by email, we cannot say that there is no guideline in place – just that we were unable to retrieve this information, rather than assume that this indicates an absence of a national guideline.
2. I have re-worded this point to explain it more clearly. Since both representatives from Bangladesh and Palau explained that whilst they formally recommend practitioners to refer to WHO guidelines, they have not developed and published guidelines that adapt WHO guidelines to the national contexts. Therefore additional quality appraisal and analysis of these guidelines would just duplicate appraisal of WHO guidelines. We cannot determine any additional information about their domestic influenza guidance.
3. Thank you – we have gladly expanded on this! We have discussed the normative clinical guideline development process. We have expanded on the point about the reality of the mismatch between clinical guideline recommendations and the domestic situation in terms of antiviral licensing, availability and access.

I have made very minor grammatical corrections in addition on final review.

References

Since original resubmission, we have added reference [41] for the latest 2024 WHO influenza guidelines.

We have also added [42] and [43] in the conclusion. We felt on reflection of yours and reviewer 1’s comments it was important to provide a stronger conclusion that describes gold standard clinical guideline development and builds on recent calls for regional cooperation in tackling infectious diseases in the interests of global health security.

7. PLOS authors have the option to publish the peer review history of their article ([what does this mean?](https://track.editorialmanager.com/CL0/https:%2F%2Fjournals.plos.org%2Fglobalpublichealth%2Fs%2Feditorial-and-peer-review-process%23loc-peer-review-history/1/010f019511eb2add-56bc6c6f-9c2b-4bcf-9252-bc00d3e81588-000000/MdeDg-lgc1Z64z1uBRhW2uoi4ze5ijCyG5jVux-Wxg0=198)). If published, this will include your full peer review and any attached files.


**Do you want your identity to be public for this peer review?** If you choose “no”, your identity will remain anonymous but your review may still be made public.


For information about this choice, including consent withdrawal, please see our [Privacy Policy](https://track.editorialmanager.com/CL0/https:%2F%2Fwww.plos.org%2Fprivacy-policy/1/010f019511eb2add-56bc6c6f-9c2b-4bcf-9252-bc00d3e81588-000000/B36dPrvYOs1AgsQApAkMeITzkSHogTvkLK4xly1RfaA=198).

Reviewer #1: No

Reviewer #2: **Yes:** Sharmistha Mishra
